# The Calcium Channel C-Terminal and Synaptic Vesicle Tethering: Analysis by Immuno-Nanogold Localization

**DOI:** 10.3389/fncel.2017.00085

**Published:** 2017-03-30

**Authors:** Robert H. C. Chen, Qi Li, Christine A. Snidal, Sabiha R. Gardezi, Elise F. Stanley

**Affiliations:** Laboratory of Synaptic Transmission, Krembil Research Institute,Toronto, ON, Canada

**Keywords:** presynaptic, calcium channel, synaptic vesicle, tether, tethering, CaV2.2, transmitter release, C-terminal

## Abstract

At chemical synapses the incoming action potential triggers the influx of Ca^2+^ through voltage-sensitive calcium channels (CaVs, typically CaV2.1 and 2.2) and the ions binds to sensors associated with docked, transmitter filled synaptic vesicles (SVs), triggering their fusion and discharge. The CaVs and docked SVs are located within the active zone (AZ) region of the synapse which faces a corresponding neurotransmitter receptor-rich region on the post-synaptic cell. Evidence that the fusion of a SV can be gated by Ca^2+^ influx through a single CaV suggests that the channel and docked vesicle are linked by one or more molecular tethers (Stanley, 1993). Short and long fibrous SV-AZ linkers have been identified in presynaptic terminals by electron microscopy and we recently imaged these in cytosol-vacated synaptosome ‘ghosts.’ Using CaV fusion proteins combined with blocking peptides we previously identified a SV binding site near the tip of the CaV2.2 C-terminal suggesting that this intracellular channel domain participates in SV tethering. In this study, we combined the synaptosome ghost imaging method with immunogold labeling to localize CaV intracellular domains. L45, raised against the C-terminal tip, tagged tethered SVs often as far as 100 nm from the AZ membrane whereas NmidC2, raised against a C-terminal mid-region peptide, and C2Nt, raised against a peptide nearer the C-terminal origin, resulted in gold particles that were proportionally closer to the AZ. Interestingly, the observation of gold-tagged SVs with NmidC2 suggests a novel SV binding site in the C-terminal mid region. Our results implicate the CaV C-terminal in SV tethering at the AZ with two possible functions: first, capturing SVs from the nearby cytoplasm and second, contributing to the localization of the SV close to the channel to permit single domain gating.

## Introduction

The fusion of synaptic vesicles (SVs) at the active zone (AZ) of classical fast-transmitting presynaptic terminals is gated by the influx of calcium ions (Ca^2+^) through voltage-gated calcium channels (CaVs) at the presynaptic membrane (Katz and Miledi, 1965; Llinás et al., 1992). The finding that Ca^2+^ influx through a single CaV could gate the fusion of a SV was the basis of the prediction that the channel and vesicle must be in close proximity and linked by a molecular tether (Stanley, 1993, 1997, 2016). More recent studies suggest that CaV-SV tethering may be complex and involve linkages of various lengths that presumably play different roles in the vesicle cycling and release process (see below). Although there has been considerable speculation on the molecular nature of these SV to CaV links, as yet no general consensus has emerged.

The AZ appears deceptively simple in standard electron micrographs, but novel technological and preparatory methods are revealing its architectural complexity. Early platinum-shadowed freeze-etched images of various presynaptic terminals revealed a network of cytoplasmic filaments that linked SVs to each other and to the AZ surface membrane (Landis et al., 1988; Hirokawa et al., 1989) including long, >100 nm, AZ surface membrane to SV fibrous structures (Hirokawa et al., 1989). Recently such SV-AZ fibers have been imaged in more detail by means of a variety of electron microscopy-based methods (Harlow et al., 2001; Siksou et al., 2007; Fernández-Busnadiego et al., 2013; Wong et al., 2014; Cole et al., 2016). At least two main types have been identified: a single, ‘long tether’ link (∼>45 nm) and multiple ‘short tethers’ (Fernández-Busnadiego et al., 2013; Wong et al., 2014; Cole et al., 2016). A simple hypothesis to account for these types of tethers is that the SV is initially snared, or ‘grabbed’ by the long tether which serves to guide the SV to the surface membrane where it is then ‘locked’ in place and within range of the Ca^2+^ influx of CaVs by the shorter links (Fernández-Busnadiego et al., 2013; Wong et al., 2014; Cole et al., 2016).

The finding that proteins associated with SV docking and fusion co-precipitate with presynaptic-type CaVs in biochemical analyses (Bennett et al., 1992; Yoshida et al., 1992; Seagar and Takahashi, 1998) led to early suggestions of a molecular association and were followed by analysis of the ‘synprint (synaptic protein interaction site) region’ within the CaV cytoplasmic loop between the II and III domains (II–III loop) (reviewed in Sheng et al., 1998). While such a connection could conceivably contribute to the short tether type because of maximum extension of the II–III loop backbone (∼60 nm max), they are unlikely to account for the long tethers filaments, which may be up to 190 nm in length (Wong et al., 2014).

We set out to explore the hypothesis that the CaV2.2 C-terminal is, or contributes to, the long tether. This idea was based on several published experimental findings and theoretical predictions. Residual SVs, imaged by standard electron microscopy after flushing free cytoplasm and SVs from the nerve terminal lumen of chick brain synaptosomes, were attached to AZs by thin filaments of up to 190 nm (Wong et al., 2014). With over 600 amino acids (AA) and almost entirely devoid of secondary structure (according to prediction software), the channel C-terminal tail could, at least in theory, explain such structures (Wong et al., 2014). An association of C-terminals with these long SV tethers was greatly strengthened by our identification, using a novel cell-free binding assay (SV-pull down, or SV-PD; Wong et al., 2013), of an SV binding site just proximal to the channel C-terminal tip (Wong et al., 2014) and more recently of an SV binding motif within this region (Gardezi et al., 2016).

**FIGURE 1:**
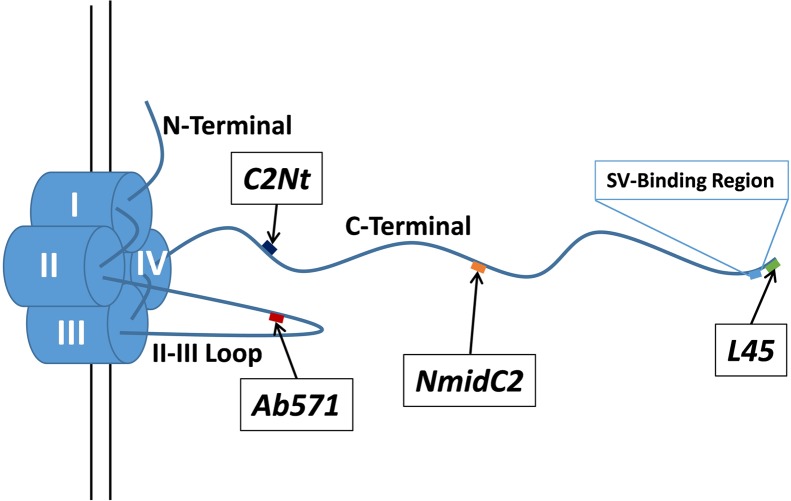
**Schematic of the chick CaV2.2 α_1_ subunit intracellular domains and the locations of the antigenic targets for the antibodies used in this study.** C2Nt, NmidC2, and L45 antibodies are targeted against the C-terminal tail at different distances from the channel pore (diagram approximately to scale based on the polypeptide backbone) while Ab571 is targeted against a point approximate half way along the II–III loop. The previously described SV-binding region is located near the distal tip of the C-terminal tail (see text).

To test the hypothesis that the channel C-terminal contributes to long cytoplasmic SV tethers, we addressed two main questions: whether the tip of the presynaptic calcium channel C-terminal contacts tethered SVs, and whether the C-terminal mid-region is located in the cytoplasmic gap between the surface membrane and the tethered SV. These questions were addressed in chick synaptosomes, to complement our previous biochemical and structural studies, by a novel immunogold labeling protocol with conventional transmission electron microscopy imaging. We localized various regions of the calcium channel intracellular domains using four antibodies (**Figure 1**): L45, a cocktail of L4569 and L4570 that were raised against the same C-terminal tip peptide (Khanna et al., 2006); Ab571, directed against the synprint region of the channel II–III loop (Li et al., 2004); and two new antibodies, NmidC2 and C2Nt, raised against peptides replicating mid and proximal-third regions of the C-terminal, respectively. Statistical analyses supported the hypothesis that the CaV C-terminal can extend from the AZ and contact SVs within the 200 nm peri-AZ cytoplasmic space.

## Materials and Methods

### Synaptosome (SSM) Ghost Tissue Preparation

Synaptosome ghost preparation was based on the protocol established for isolating SVs from SSMs (Whittaker et al., 1964) with some modifications. For each individual EM sample, the whole brains of 4–5 E15–E17 chicks were dissected out. All subsequent steps were either done on-ice or at 4°C. The brains were homogenized together in homogenization buffer (“HB”: 320 mM sucrose, 10 mM HEPES at pH 7.4, 1 mM EGTA, 1 mM phenylmethylsulfonyl fluoride and protease inhibitor cocktail (Sigma-Aldrich, St. Louis, MO, USA) at 1:200) with 10 strokes of a Dounce homogenizer (Thomas Scientific, Swedesboro, NJ, USA). The homogenate was centrifuged for 15 min at 1000 × *g*. The resulting supernatant was further centrifuged twice for 35 min at 200 000 × *g* and the pellet was resuspended in HB after each centrifugation. The sample was then passed six times through a 22.5-gage needle and prior to being loaded onto a 0.8 M/1.2 M discontinuous sucrose gradient and centrifuged for 1.5 h at 100 000 × *g* in a swing bucket rotor allowing the centrifugation to end without braking. The SSMs found in the brown-colored layer of the 0.8 M/1.2 M sucrose interface were recovered, resuspended in HB, and the synaptosomes were recovered in a pellet after centrifugation at 2000 × *g* for 30 min.

To generate synaptosome ghosts, we resuspended the synaptosome pellet in an osmotic-rupture buffer (“ORB”: 50 mM Na HEPES at pH7.4, <10 nM free CaCl with 1 mM EGTA) and recentrifuged for 30 min at 2000 × *g*. The pellet was resuspended in HB diluted to 0.2 M of its sucrose content, passed six times through a 22.5-gage needle, and then loaded onto a 0.4 M/0.6 M/0.8 M/1.0 M discontinuous sucrose gradient. This gradient was centrifuged at 100 000 × *g* in a swing bucket rotor with brakes disabled for 1.5 h and left overnight in the centrifuge. The purified SSM ghosts layer at the 0.8 M/1.0 M interface was diluted in ORB and divided into the desired number of EM samples. Each sample was then centrifuged into a pellet at 20 000 × *g* for 30 min.

### SSM Ghost Passive Diffusion Antibody Labeling

Synaptosome ghost pellets were undisturbed and fixed for 1 h at room temperature with 50 uL of Fix Solution #1 (4% paraformaldehyde, 0.1% glutaraldehyde, 0.1 M cacodylate buffer pH 7.2) followed by two gentle rinses with 100 uL of 150 mM Tris-HCl (pH7.2) for 15 min each to saturate residual aldehydes. The pellets were resuspended with 50 uL of a non-selective antibody binding site blocking solution (1.2 mg/mL of goat serum in 20 mM Tris-HCl pH 7.2) for 30 min on ice. Primary antibody (L4569 from Khanna et al., 2006; or 1 mg/mL non-specific rabbit IgG from Jackson ImmunoResearch, West Grove, PA, USA) was added at 1:100 dilution and the mixture was left on a rocker overnight at 4C. The following morning, samples were centrifuged at 20 000 × *g* for 1 h and the resulting pellet was gently rinsed twice with 100 uL of 20 mM Tris-HCl. The pellet was then resuspended in 100 uL of 20 mM Tris-HCl and centrifuged again at 20 000 × *g* for half an hour. Pellets were then resuspended in 50 uL of 20 mM Tris-HCl with 1:100 6 nm colloidal gold goat anti-rabbit secondary antibody (Electron Microscopy Sciences, Hatfield, PA, USA). Samples were then incubated for 2 h at room temperature before being centrifuged for 30 min at 20 000 × *g*. Pellets were then rinsed gently twice with 1 mL of 20 mM Tris-HCl and then resuspended. After centrifugation at 20 000 × *g* for 30 min, pellets were rinsed twice with 100 uL 0.1M cacodylate buffer (Electron Microscopy Sciences, Hatfield, PA, USA). Samples were then fixed again as described below.

### SSM Ghost Antibody Cryoloading

Cryoloading is described in detail in a previous publication (Nath et al., 2014). SSM ghost pellets were resuspended in sucrose/EDTA/Tris buffer (“SET”: 320 mM sucrose, 1 mM EDTA, 5 mM Tris at pH 7.4) with 5% DMSO at room temperature. Primary antibodies were added such that the final antibody concentration was 1:50. Samples were then frozen slowly by enclosing them in a parafilm-wrapped Styrofoam freezer box stuffed with lab diapers prior to being put into a −80°C freezer. Samples were left in the freezer either overnight or over a series of days (no changes in labeling efficacy were observed in relation to the number of days left frozen), and then were thawed quickly in a 37°C incubator for 2.5 min. Ice-cold ORB was added at 10 times the volume of each sample to rupture any resealed SSM ghosts and to flush out unbound primary antibody. Ghosts were then re-cryoloaded with Nanogold^®^ goat anti-rabbit secondary Fab fragments (Nanoprobes, Yaphank, NY, USA) at a 1:50 concentration in SET buffer and 5% DMSO in the same fashion as was done for cryoloading of the primary antibodies. After cryoloading with the secondary Fab fragments, ghosts were again thawed and ruptured with ice-cold ORB to flush out unbound secondary Fab fragments prior to being centrifuged at 20 000 × *g* for 1 h at 4°C. Ghost pellets at this point were generally ∼1 mm^3^ in size and were then fixed again described below.

### SSM Ghost Fixation

All subsequent steps were done at room temperature and samples were left in the dark during incubations. SSM ghosts were fixed as pellets using 200 uL of Fix Solution #2 (2% paraformaldehyde and 2% glutaraldehyde in 0.1 M cacodylate buffer pH 7.2). Without disturbing the pellets, the supernatant was exchanged for 500 uL of a 150 mM pH 7.4 tris buffer to quench the fixatives and allowed to sit for 30 min. The supernatant was then exchanged for 500 uL of 0.1M cacodylate buffer (pH 7.4) twice and allowed to sit for 30 min after each exchange. The samples were then left overnight to remove as much tris as possible.

### EM Preparation and Imaging

The sample pellets were stained with 2% osmium tetroxide (Electron Microscopy Sciences, Hatfield, PA, USA) in 0.1 M cacodylate buffer for 1 h. The pellets were washed twice for 10 min each with 0.1 M cacodylate buffer and then twice for 10 min each with distilled water. The pellets were then stained *en bloc* with 1% uranyl acetate (Electron Microscopy Sciences, Hatfield, PA, USA) for 1 h. After three 10 min washes in distilled water, the pellets were put through a series of 30, 50, 70, 90, and 100% ethanol washes of 10 min each. The pellets were then put through a series of 1:3, 1:1, and 3:1 dilutions of Spurr’s resin (Electron Microscopy Sciences, Hatfield, PA, USA) in ethanol, and left in pure Spurr’s resin overnight. The next day, the resin was replaced with fresh resin twice in ∼5 h intervals before the samples were placed into a 60°C oven overnight to cure. 100 nm sections of each sample were cut using a Leica EM UC6 ultramicrotome (Concord, ON, Canada) and placed onto copper grids (Electron Microscopy Sciences, Hatfield, PA, USA).

Sections were examined using a Hitachi (Tokyo, Japan) H7000 (for passive diffusion labeling experiments) or HT7700 transmission electron microscope (all subsequent experiments) at 75 kV or 80 kV, respectively (imaging done at Cell & Systems Biology Imaging Facility, University of Toronto, Toronto, ON, Canada). The top portions of pellets (where the density of sample material was low enough to easily differentiate synapses from structures randomly abutting each other from centrifugation) were scanned at low magnification (50K× magnification) for ghosts with visibly attached post-synaptic scabs. Higher magnification images were then taken of these ghosts to be used for quantification purposes. Ghosts labeled with 6 nm colloidal gold secondary antibodies using passive diffusion (such as **Figure 1**) were imaged at 300K× magnification. Ghosts labeled with L45 or Ab571 antibodies were imaged at 500K× magnification. Ghosts labeled with NmidC2 antibodies prior to December 2015 were imaged at 500K×, while those afterward as well as C2Nt labeled ghosts were imaged at 530K×. The change in high magnification imaging was due to an upgrade to the TEM software beyond our control. Despite their small size, nanogold particles were generally readily identified on the electron micrograph. However, because of their intense black stain, some confusion could occur at cross-sectioned lipid bilayers. Fortunately, the antibodies must be excluded from such regions and hence, false positive or negative identification is unlikely.

### Compartment Analysis

Images at 500K× or 530K× magnification (where nanogold particles are readily visible) were subdivided by hand using ImageJ (NIH, Bethesda, MD, USA) into separate compartments (**Figure 4A**). The combined area for each compartment from each image for each condition were quantified using ImageJ, and the total number of gold particle clusters within these compartments were tallied. The compartment area vs. number of gold clusters were plotted on a scatterplot in GraphPad Prism 6 (La Jolla, CA, USA). 95% prediction bands were calculated from the control data using GraphPad Prism 6.

### Antibodies

The presynaptic marker SV2 antibody (DSHB, Iowa City, IA, USA) and Purkinje neuron marker Calbindin (D28K from Synaptic Systems, Gottingen, Germany) are commercially available antibodies.

Rabbit polyclonal antibodies that were generated in house and that have been characterized previously included L4569 and L4570 (Khanna et al., 2006). These two antibodies were generated in separate rabbits but with the same CaV2.2 C-terminal tip antigenic peptide. An equal volume combination of L4569 and L4570 was termed L45 and was used for labeling in this study. Ab571 was raised against a peptide from the synprint region of the chick CaV2.2 II–III loop (Li et al., 2004) and has been characterized extensively (see text).

Two new antibodies were generated for this study. An *RPHPMHLYEYSLER* peptide, replicating a sequence in the mid region of the chick CaV2.2 C-terminal (**Figure 1**) tail was used to generate NmidC2. An *LSPKNLDLLVTPHK* peptide, replicating a sequence within the proximal third of the chick CaV2.1 C-terminal tail (**Figure 1**; Chick CaV2.1 full length sequence submitted to Genbank) was used to generate C2Nt. KLH-conjugated immunogenic peptides (NmidC2 peptide made by SPARC BioCentre, Hospital for Sick Children, Toronto, ON, Canada; C2Nt peptide made by Biomatik, Cambridge, ON, Canada) were used for antibody generation in white rabbits (immunization and bleeding done by the animal facility of the Division of Comparative Medicine, University of Toronto, Toronto, ON, Canada based on protocols established in Harlow and Lane, 1988). Five hundred microgram of immunogenic peptides (diluted to 1 mg/mL in PBS) were injected into rabbits with Freund’s Complete Adjuvant as the primary immunization while 250 ug of peptides along with Freund’s Incomplete Adjuvant was injected as subsequent boosts. Rabbits were bled 2 weeks after an immunization and rabbits were re-inoculated with boosters a week after being bled.

Serum with the NmidC2 antibody was affinity-purified using a SulfoLink Immobilization Kit (#44999 from ThermoFisher, Waltham, MA, USA). The affinity-purified NmidC2 was used only for EM experiments that did not involve antibody-blocking. NmidC2 serum was used directly for all other experiments. C2Nt experiments were all carried out using serum.

### Western Blotting

Western blotting was done using conventional methods (Wong et al., 2013). Protein were mixed with Laemmli sample buffer (Bio-Rad, Hercules, CA, USA) with 5% β-mercaptoethanol and boiled for 5 min at 100°C prior to being loaded onto 10% SDS-PAGE gels and run at 120 V. Gels were then transferred onto PVDF membrane (Bioshop, Burlington, ON, Canada) and blocked for 1 h with 5% milk in TBST (10 mM Tris-HCl pH 8.0, 150 mM NaCl, 0.1% Tween-20; Bioshop, Burlington, ON, Canada). Blots were incubated overnight at 4°C with primary antibody (at 1:1000 for blots of fusion protein, and 1:200 for all other blots) in 5% milk in TBST. Blots were then washed 3× 10 min with TBST prior to incubation for 1 h at room temperature with secondary goat anti-rabbit antibodies (at 1:3000; Jackson Immunoresearch, West Grove, PA, USA) conjugated with horseradish peroxidase in 5% milk in TBST. Blots were washed again 3x 10 min with TBST and treated with ECL (GE Healthcare, Chicago, IL, USA). Chemiluminescence was visualized using a ChemiDoc XRS System (Bio-Rad, Hercules, CA, USA).

### Dot Blotting

Nitrocellulose membrane was cut into squares and each square was marked with a circle near each corner (i.e., four circles in total) using pencil. One microliter of fusion protein was applied within each circle and allowed to air dry for approximately 15 min. Two different fusion proteins were used at different concentrations. The C3strep fusion protein (Wong et al., 2013) replicated a distal region of the CaV2.2 C-terminal and contained the antigenic region for the L45 antibodies but not the antigenic region for the NmidC2 antibodies. Five nanogram of C3strep was applied to the top left hand circle of a square while 50 ng was applied to the top right hand circle of a square. The other fusion protein used was C1-2strep which replicated a more proximal region of the CaV2.2 C-terminal that contained the antigenic region for the NmidC2 antibodies but not the antigenic region for the L45 antibodies. Because the C1-2strep fusion protein was eluted from the beads used for its purification by heat alone, the amount of C1-2strep fusion protein applied to the bottom two circles of the dot blots is unknown. However, the left hand circle contains C1-2strep at a 10x lower amount than the right hand circle. Blots were blocked for 1 h at room temperature in 5% milk in TBST. Blots were then incubated overnight at 4°C with either L45 or NmidC2 antibody sham-blocked or blocked with various concentrations of their respective antigenic peptides (see below for antibody blocking). The next day, the blots were washed three times for 10 min each with TBST. Blots were then incubated for 1 h at room temperature with goat-anti-rabbit-HRP secondary antibodies (same as above for western blotting) at 1:3000 dilution in TBST with 5% milk. After another three rounds of 10 min washes in TBST, blots were incubated with ECL for 5 min and then imaged on the ChemiDoc XRS System.

### Fluorescent Immunostaining

Fluorescent immunostaining was done either on chick cerebellar slices or chick ciliary ganglion (CCG; Stanley and Goping, 1991) cells from E15 embryonic chicks. Immunostaining was conducted as described (Li et al., 2004) and at room temperature, unless otherwise stated. Chick cerebella were dissected and fixed in 2% paraformaldehyde and a series of 30% (2 h), 20% (2 h), and 10% (overnight) sucrose in PBS (1x phosphate buffered saline pH 7.4; ThermoFisher, Waltham, MA, USA). The cerebella were then embedded in M1 Freezing Medium (ThermoFisher, Waltham, MA, USA) and sectioned into 15 μm thick slices using a Leica CM3050 S cryostat. CCGs were fixed (150 mM cyclohexylamine, 20 mM EGTA, 20 mM MgCl_2_, 20 mM PIPES, and 2% paraformaldehyde, pH 6.5) on glass coverslips for 45 min. Fixatives were then quenched (150 mM Tris, 20 mM MgCl_2_, 20 mM NaN_3_, pH 7.4) for 30 min before being washed for with Ab Buffer (500 mM NaCl, 10 mM MgCl_2_, 10 mM NaN_3_, 20 mM Tris, 0.1% BSA, pH 7.2) for 30 min. Both CCGs and cerebellar slices were blocked with 5% donkey serum in Ab Buffer for 45 min. Incubation with primary antibody (diluted in pH 6.8 Ab Buffer) was at 4°C overnight. Tissue was then washed 3x 5 min with pH 7.2 Ab Buffer prior to being blocked again with 5% donkey serum in Ab Buffer for 45 min. Tissue was then incubated with 1:100 secondary (Jackson ImmunoResearch, West Grove, PA, USA) donkey anti-mouse conjugated to AlexaFluor 594 and/or donkey anti-rabbit conjugated to FITC for 1 h. After another 3x 5 min wash with Ab Buffer, tissue was mounted using Vectashield (Vector Laboratories, Burlingame, CA, USA) and imaged using an Axioplan 2 microscope (Zeiss, Oberkochen, Germany).

### Antibody Blocking

NmidC2 antibody was incubated with an equal volume of 40 μM of either peptide corresponding to the NmidC2 antigenic site for 2 h on ice (NmidC2 peptide made by SPARC BioCentre, Hospital for Sick Children, Toronto, ON, Canada; other peptides made by Biomatik, Cambridge, ON, Canada). A combination of two peptides corresponding to the L45 antigenic site was used as a control. The final concentration of antibody was adjusted for peptide addition to be approximately the same as used for our standard biochemical procedures.

## Results

### Development of an Immunogold Labeling Method for Synaptosomes Ghosts

**FIGURE 2:**
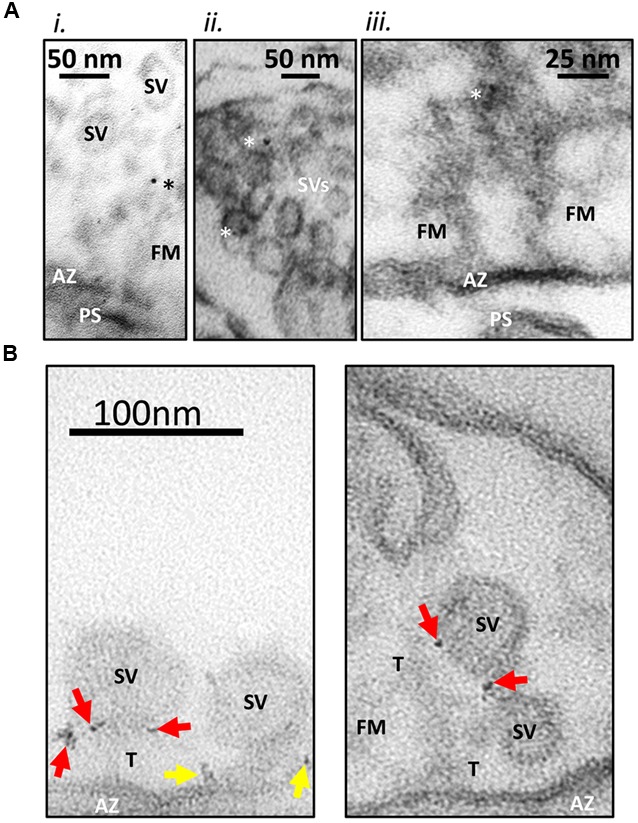
**Immunogold labeling method. (A)** Standard immunogold labeling method. Fresh synaptosomes were ruptured by osmotic shock, fixed and incubated with L4569, an anti-CaV2.2-C-terminal tip antibody. SSMs were prepared and sectioning for EM after exposure to colloidal gold (6 nm) conjugated secondary antibody. (i) Gold particle in the SV tether zone. (ii) Two SVs with associated gold in the peri-AZ region. (iii) Active zone (AZ) region of an SSM, opposing a short post-synaptic scab (PS) devoid of SVs but projecting fibrous material (FM; fibrous material linking the SV to the surface membrane are labeled as tethers, (T). Three dark spots were identified as a triplet of gold particles. These are located in the fibrous net ∼75 nm from the AZ. The images suggest that gold particles are associated both with SVs and fibrous material near the AZ. Particles were rarely seen at the surface membrane. Ghosts were imaged at 300K×. Gold particles denoted with asterisks. **(B)** New nanogold cluster labeling method. SSM ghosts were cryoloaded with L45 antibody against the CaV2.2 C-terminal tail to facilitate access to the intra-ghost epitopes. The ghosts were then cryoloaded with the Nanogold^®^-tagged goat anti-rabbit Fab fragment secondary and were imaged as in **(A)** but at 500K×. The small gold particles were observed most often as small clusters (arrows) which greatly facilitated the localization of the primary antibody. In these examples the nanogold clusters are frequently associated with the tethered synaptic vesicles (SVs; red arrows) or the AZ surface membrane (yellow arrows). Other labels are as in **(A)**.

In earlier studies (Khanna et al., 2006), we developed and characterized two rabbit polyclonal antibodies (L4569 and L4570) directed against the same polypeptide that replicated the distal tip of the CaV2.2 (**Figure 1**). In our preliminary immunogold labeling attempts, the primary antibody was added to the synaptosome ghost samples and, after an extensive wash protocol, L4569 binding was chased with a secondary antibody tagged with 6 nm colloidal gold. We assumed that a significant fraction of the synaptosome surface membranes would be incompletely resealed, permitting access of the antibodies to intra-terminal structures by passive diffusion. However, this method had limited success. While occasional gold particles were observed with SVs and on filaments that radiate from the AZ into the synaptosome lumen (**Figure 2A**), the labeling efficacy was insufficiently high to quantify the data.

We therefore set out to devise a more effective immunogold labeling protocol, addressing two specific methodological concerns. The first concern was that a significant fraction of the synaptosomes may have resealed after the osmotic shock step, occluding antibody access to the luminal face of the AZ. This concern was solved by freeze-thaw-loading of the antibodies into the resealed synaptosomes using a protocol that we term cryoloading as described recently in detail (Nath et al., 2014). To improve labeling efficiency, we used a cocktail of L4569 and L4570, termed here L45, that is in effect a more heterogeneous polyclonal antibody than either alone.

Our second concern was that the colloidal gold particles, which are attached to the secondary antibody solely by electrostatic binding (Faulk and Taylor, 1971) might dissociate during the labeling protocol and thus reduce tagging efficiency. Rather than attempt to quantify this possibility, we avoided it by switching to anti-rabbit Fab fragments tagged with a single 1.4 nm nanogold particle by covalent binding (see Materials and Methods). The use of Fab fragments rather than a complete secondary antibody has the added advantage of reducing the total distance from the primary antibody binding epitope to the location of the gold particle. We were concerned initially that the small size of this gold particle might be difficult to visualize reliably in electron micrographs. Fortunately, the only truly electron-dense presynaptic regions were cross-sectioned lipid bilayers that are very unlikely to exhibit significant antibody tagging. Indeed, the anticipated disadvantage of imaging small gold particle was far more than compensated by the finding that primary antibodies were generally tagged by multiple Fab fragments resulting in a ‘nanogold cluster’ (**Figure 2B**). Hence, we improved not only the efficacy of staining but also our confidence in its identification with our gold labeling method.

We scored staining as positive if two or more gold particles (identified as obviously black or near-black dots) were located within a radius of 5 nm, a staining pattern that was termed a ‘nanogold cluster.’ In both test and control images, we occasionally observed a synaptosome with many, seemingly randomly distributed nanogold particles. These were attributed to passive entrapment of the nanogold Fab secondary by membrane resealing prior to the final wash step and were excluded from further analysis.

### Visualization of Tethers and Synaptic Vesicles in Synaptosome Ghosts

As reported previously (Wong et al., 2014), electron micrographs of synaptosome ghosts revealed fibrous projections radiating from the presynaptic AZ regions. ∼40 nm diameter SVs were also observed that were linked to the surface membrane by fiber-like ‘tethers’ (**Figure 2B**). Our subsequent experiments were carried out to test if the channel C-terminal was related to these tether structures.

### Localization of the CaV2.2 Channel C-Terminal Tip

**FIGURE 3:**
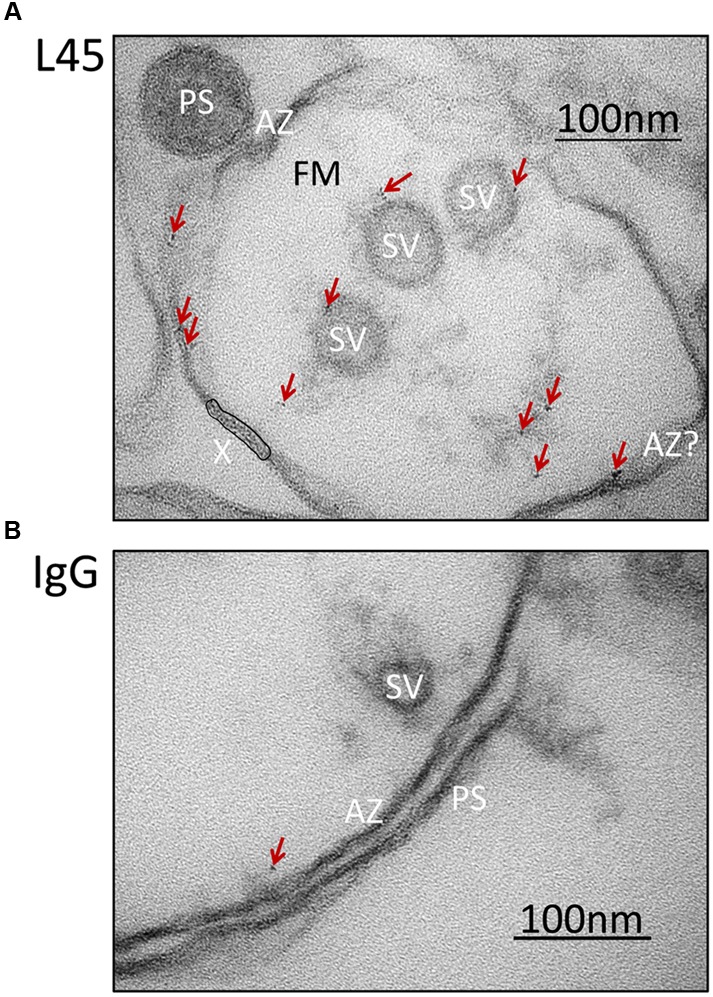
**Synaptosome ghosts immunogold labeled with an antibody against the tip of the CaV2.2 C-terminal.** SSM ghosts were first cryoloaded with primary L45 **(A)** or non-specific rabbit IgG control **(B)** as in **Figure 2B**. With L45-cryoloaded ghosts nanogold particle clusters (denoted by crimson arrows) were observed on SVs and active zone membrane (AZ). Ghosts that were cryoloaded with control rabbit IgG (representative image in bottom panel) exhibited fewer gold particle clusters (crimson arrow) and these were not associated with a particular subcellular structure. Labels as in **Figure 2A**. AZ?, Likely, but unconfirmed active zone; X, region of membrane with false-positive, nanogold-like dark spots (see Materials and Methods, EM Preparation and Imaging).

Synaptosome ghosts were treated with the L45 antibody cocktail followed by Fab-nanogold secondary, as discussed above, with paired rabbit IgG controls. Nanogold clusters were observed in both L45 and control ghosts, but at a higher frequency in the former (control 0.36 ± 0.15 clusters/ghost, L45 3.17 ± 0.84; *p*_t-test_ ≤ 0.005). As assessed by eye, the few nanogold clusters in the controls were not localized with any particular structure (**Figure 3B**), but with L45 they were frequently associated with both the surface membrane and tethered SVs (**Figure 3A**). A few nanogold clusters were located at the contact point of tethers with the SV (**Figures 2B**, **3**).

### Subcellular Organelle-Association of L45 Antibody Nanogold Cluster

**FIGURE 4:**
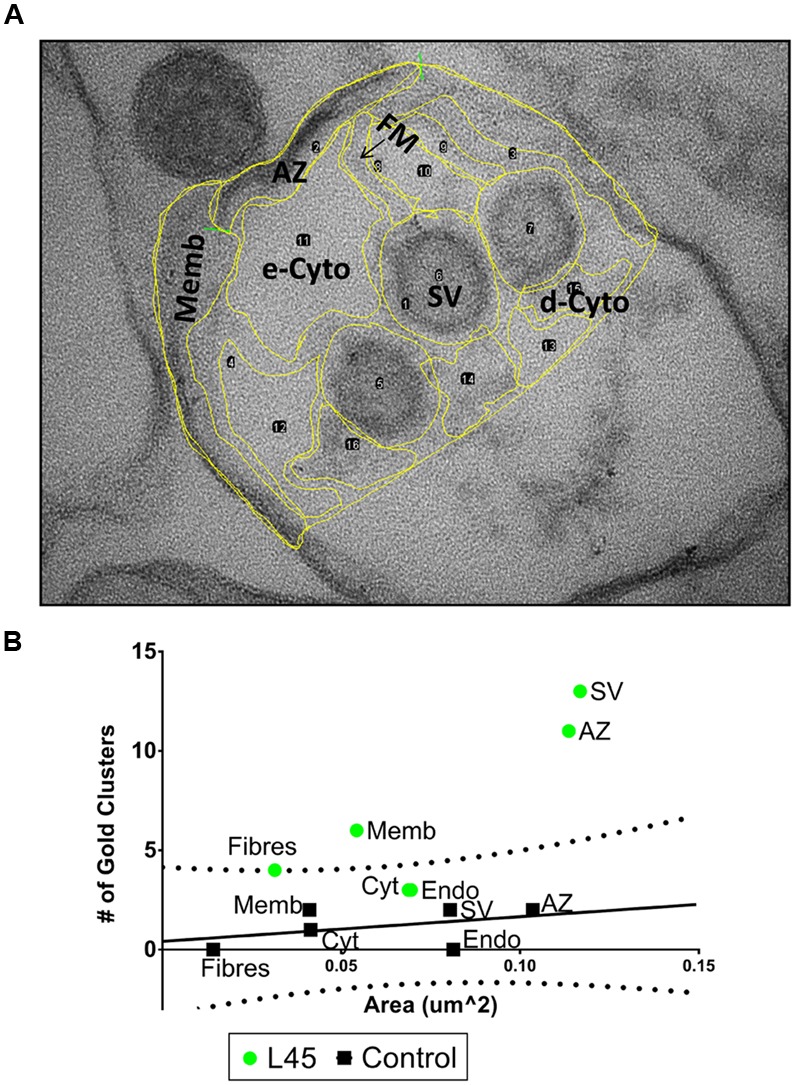
**Compartment analysis of immunogold labeling in L45-cryoloaded ghosts. (A)** An example of regional compartments identified by structural markers in a synaptosome ghost section including: *AZ*, active zone membrane; *Memb*, non-AZ plasma membrane; *FM*, filaments running from the AZ; *SV*, synaptic vesicles; *e-Cyto*, cytoplasm devoid of electron-dense structures; *d-Cyto*, cytoplasm with electron-dense structures. *Endo*, endosomes and unidentifiable membrane-enclosed organelles were not present in this image but were present in other ghosts examples. The same image as in **Figure 3A** but with a lower contrast to view yellow border line overlay. **(B)** For each ghost, we counted the number of nanogold clusters and measured the total area of each compartment. Then, for each treatment, the total number of nanogold clusters and the total area of each compartment were pooled. These values were then displayed as a scatter plot. In controls *(black squares)* the number of nanogold clusters increased approximately linearly with net compartment area, consistent with random labeling. The control data was fit with a regression line (*black line*) together with its 95% prediction bands (*dotted lines*). The L45 experimental data were plotted on the same axes (*green circles*). e-Cyto data were omitted from the plot (see text). *n* = 12 ghosts from 3 separate IgG and L45 immunolabeling experiments.

We devised a quantitative ‘compartment analysis’ method to test if the nanogold clusters were associated with particular intracellular structures. Briefly (see Materials and Methods for details), a set of digital EM images of SSM ghosts were divided by eye into visually identifiable regions or ‘compartments’ such as surface membrane, SV, dense cytoplasm, etc. (**Figure 4A**). We noted the number of nanogold clusters in each compartment and calculated the total net area of the ghost occupied by that compartment type. The cluster counts and area occupied for each compartment were then summed for each treatment set. The pooled data was then plotted as the number of gold particle clusters against the total area viewed for each compartment (**Figure 4B**). Our null-hypothesis was that for background, random staining ‘the number of particles should be linearly related to the summed compartment area.’ The alternative hypothesis for compartment-specific staining was: ‘increased test antibody binding will result in an enhanced number of nanogold clusters that will diverge from the background, linear relationship.’ Empty regions of the ghosts that were vacated during the osmotic shock were omitted from the analysis since, understandably, there was nothing for the antibodies to adhere to (indeed, these areas were devoid of nanogold clusters).

Compartment analysis of the L45 immunogold experiment was carried out blind by an investigator (EFS) that was not involved in the electron microscopy or image collation steps. Analysis of the control ghosts was consistent with the null hypothesis. Nanogold cluster frequency in each compartment type increased approximately in proportion to their respective summed areas (**Figure 4B**). The compartment particle density versus compartment area data was fit by a straight line regression and while the slope failed to reach significance (*R*^2^ = 0.178, *p* = 0.405) we could nonetheless calculate the 95% Prediction Bands. Values from a similar compartment analysis using the test antibody, L45, were then superimposed on the control plot. The L45 electron dense cytoplasm and endosome compartments fell within the 95% prediction bands, consistent with background, non-specific staining. However, the AZ fibers compartment value was just outside the 95% prediction band while the values for the AZ and non-AZ surface membrane compartments and the SV compartment were well outside. Thus, compartment analysis suggests that L45 antibody binding, and hence the CaV2.2 C-terminal tip, localizes to the AZ, the tethered SVs, the non-AZ membrane and the AZ fibrous projections.

### Localization of L45 Nanogold Cluster on Individual Tethered SVs

**FIGURE 5:**
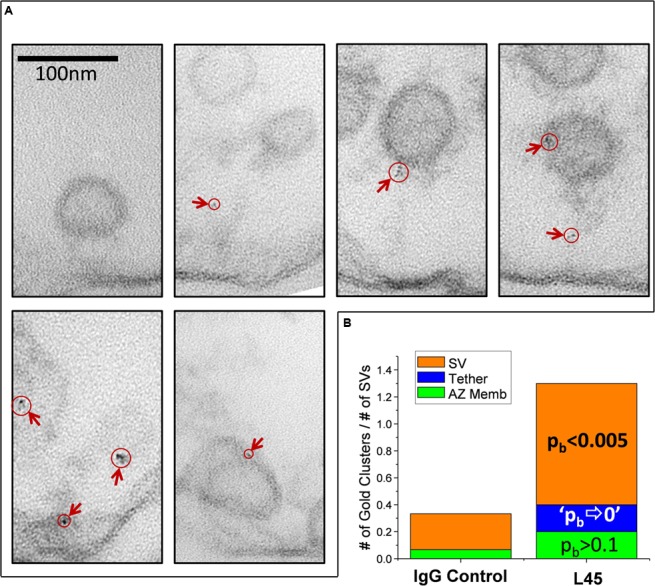
**Imaging individual L45 immunogold tethered SVs. (A)** Tethered SVs were identified for analysis at a magnification well below that required to detect nanogold. SVs were then photographed at 500K× magnification and nanogold clusters were identified off-line. Each panel shows tethered SVs with nanogold clusters identified by circles and arrows. **(B)** Analysis of nanogold cluster location on individual tethered SVs. Each tethered-SV nanogold cluster was attributed one of three regions as described in the text: the SV itself, the tether or the AZ membrane region. The number of gold clusters were normalized to the total number of individual tethered SVs analyzed for the IgG controls or L45 (*n* = 15 and 10, respectively). The significance of the L45 counts was compared to controls using the binomial test. The absence of any nanogold clusters in the tether region of the control dataset resulted in the approximation of *p*⇒0.

Compartment analysis suggests that L45 binding is associated with the fibrous projections from the AZ, which we presume include putative tethers, and also SVs. To explore this observation in detail we focused on individual tethered SVs in the L45 nanogold labeled synaptosome ghosts (**Figure 5A**). We took two precautions to ensure that sampling was unbiased. First, when scanning through the ghost sections on the EM, we operated at a magnification of 50K×. This was a sufficiently high magnification to spot AZs but too low to observe the nanogold clusters. We then photographed every AZ at high magnification (500–535K×; the small increase in gain occurred when the facility TEM system was updated) and included every tethered SV detected in our analysis whether or not gold labeling was detected.

For analysis, each tethered SV was divided into three regions for analysis: the SV itself, the tether-region AZ, and the intervening tether. These were defined as follows: Nanogold clusters that were within 10 nm of the SV or tether-region AZ were attributed to those structures while clusters in the intervening region were attributed to the tether itself. The number of nanogold clusters observed was normalized to the total number of tethered SVs analyzed for each treatment and the L45 treated data was compared to the IgG control by binomial (*p*_b_) analysis (**Figure 5B**). The number of nanogold clusters in the tether region near the AZ membrane was slightly increased but was not statistically significant (2 clusters/10 tethered SVs for L45 vs. 1/15 for control; *p*_b_ > 0.1). Nanogold cluster frequency was borderline increased for tethers themselves (2/10 for L45 vs. 0/15 for control; *p*_b_ ⇒ 0) but was markedly so for SVs (9/10 for L45 vs. 4/15 for control; *p*_b_ < 0.001). We also noted that 9/10 of the SVs with nanogold clusters were remote from the surface membrane with an average displacement into the synaptosome lumen of 39.7 ± 9.0 nm. Thus, the individual tether analysis supported the compartment analysis and we conclude that the tip of the CaV2.2 C-terminal can contact tethered SVs.

### Localizing the CaV2.2 Channel Independently of the C-Terminal

Localization of the CaV2.2 C-terminal tip to tethered SVs was of particular interest since the channel pore is known to be located within the AZ surface membrane. These findings support the provocative hypothesis that the channel C-terminal spans the cytoplasmic space and makes physical contact with the SVs. An alternative, albeit unlikely, hypothesis was that the channel transmembrane pore can also be located on the SV itself and, hence, the L45 antibody was identifying this CaV2.2 subset. This idea was tested by nanogold localization of CaV2.2 using Ab571, an affinity purified, polyclonal rabbit antibody raised against a peptide within the synprint region of the channel II-III cytoplasmic loop (Li et al., 2004). Ab571 has been used to localize CaV2.2 channels in numerous studies and has been characterized extensively by biochemical, immunocytochemical, and mass spectroscopic methods (Li et al., 2004; Khanna et al., 2007a; Gardezi et al., 2010).

**FIGURE 6:**
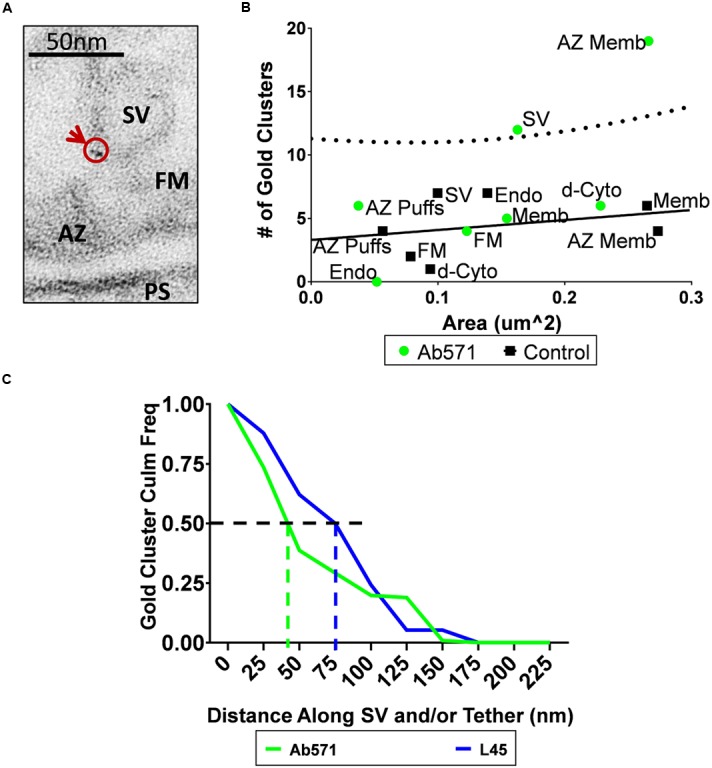
**Immunogold localization of the CaV2.2 channel by an antibody against its II–III loop. (A)** Immunostain with Ab571 showing a nanogold cluster associated with an SV located close ( ≤ 65 nm) to the active zone. **(B)** Compartment analysis of Ab571 immunogold labeled ghosts, as in **Figures 4A,B**. Dotted line denotes the 95% prediction limit for a regression fit to the control IgG data (*n* = 19; *black squares*) with the Ab571 data overlain (*n* = 19; *green circles*). **(C)** Inverse cumulative frequency histogram of the distance of each gold cluster from the surface membrane traced along the tether for individual SVs. Data was compensated for non-specific labeling as measured with respective IgG controls and normalized to the number of ghosts analyzed (Ab571, *n* = 19; L45, *n* = 12). The color-coded vertical dashed lines identify the distance for 50% of the nanogold cluster frequency for each condition.

Compartment analysis of synaptosome ghosts immunogold labeled using Ab571 identified two structures with significant nanogold cluster labeling: the AZ and SVs (**Figures 6A,B**; see Discussion). At least at first sight, this result supported the alternative hypothesis that CaV2.2 channel pores are located on the SV itself. We reasoned that if this was the explanation for the SV-associated nanogold clusters for Ab571, as with L45, the pattern of labeling should be very similar for individual tethered SVs. We explored this idea by measuring the distance along individual tethers from the surface membrane to each nanogold cluster and compared the results with the two antibodies on a cumulative frequency histogram (**Figure 6C**). The frequency of nanogold clusters dropped off precipitously with distance along the tether for Ab571 but declined more gradually with L45. 50% of the Ab571 nanogold clusters were within 45 nm from the surface membrane but extended to 80 nm for L45. Note that these measurements are not absolute as we have a bird’s-eye view and cannot detect *z*-axis displacements within the thickness of the section. However, since this limitation is similar for all tethers examined, they are at least relative. The implications of the difference in tether lengths is that L45 labeling cannot be accounted for by SV-associated calcium channels, and interestingly, that Ab571 nanogold clusters associate with a different population of tethered SVs that are located closer to the surface membrane. The latter is discussed further below.

### Does the CaV2.2 C-Terminal Span the Gap between the Active Zone and the Tethered Synaptic Vesicle?

To test the idea that the channel C-terminal spans the cytoplasmic gap between the surface membrane and the tethered SV, we raised two antibodies against peptides from C-terminal middle regions, NmidC2 and C2Nt, for nanogold staining. Our strategy was to develop antibodies that bind to the middle region and near the base of the C-terminal with the prediction that nanogold staining would be observed in the space between the AZ and the SV.

#### NmidC2 Antibody

The NmidC2 polyclonal rabbit antibody was raised against a 14 amino acid peptide replicating a region approximately halfway along the CaV2.2 C-terminal (**Figure 1**) and was characterized by western blot and immunocytochemistry. NmidC2 identified several bands in a Western blot of ghost membrane lysate (Supplementary Figure 1A) including one above the 250 kDa marker at a similar molecular weight as the CaV2.2 band identified by Ab571. Molecular specificity was carried out by ‘dot blot’ of C-terminal fusion proteins immobilized on a transfer membrane (Supplementary Figure 1B, see Materials and Methods). As expected, NmidC2 antibody was negative while L45 was positive against a fusion protein, C3, of the distal third of the C-terminal (Wong et al., 2014). In contrast, NmidC2, but not L45, identified a second fusion protein, C1-2, of the proximal two thirds of the C-terminal that contains the NmidC2 antigenic region. Further, pre-block of NmidC2 antibody using its antigenic peptide markedly reduced the intensity of the C1–C2 dot blot. The antibody also identified the fusion proteins on Western blots (Supplementary Figure 1A).

NmidC2 immunocytochemistry at the CCG calyx-type synapse generated punctate staining along the presynaptic terminal transmitter release face (Supplementary Figure 1C) that was very similar to previously published staining using our anti-CaV2.2 antibody Ab571 (Li et al., 2004; Khanna et al., 2006, 2007a,b). This finding confirms that NmidC2 can identify the channel *in situ*.

#### C2Nt Antibody

Transmitter release at fast-transmitting synapses is gated primarily by CaV2.1 as well as CaV2.2 voltage-sensitive calcium channel types. To expand the scope of our experiments we focused on a different region of the C-terminal, one located closer to the surface membrane (132 AA from the channel transmembrane domain; **Figure 1**). In an attempt to improve our nanogold labeling yield, we developed an antibody (C2Nt) against a 14 AA peptide from the chick CaV2.1 channel, LSPKNLDLLVTPHK, in which 11 of the 14 AAs, **LS**Q**K**T**LDLLV**P**PHK** (underlined and bold face) are in common with chick CaV2.2.

C2Nt was characterized as with NmidC2. It recognized fusion proteins mimicking the C-terminal tails of both CaV2.1 and CaV2.2 that contain its antigenic sequence (Supplementary Figure 2B). C2Nt immunocytochemistry of dissociated CCG resulted in specific staining of the presynaptic terminals with the puncta-like pattern seen with NmidC2 and Ab571 (Supplementary Figure 2C).

Since the antibody was raised against a peptide that is in common with CaV2.1, we also tested the antibody on chick cerebellar slices to test for staining of Purkinje neurons, the cell type in which these channels were originally described (Llinas et al., 1989). The Purkinje neurons were identified by an anti-calbindin counterstain. In the 15 days embryonic chick, calbindin staining is limited to the Purkinje neuron cell body and proximal dendrites. C2Nt stained the surface membrane of calbindin-positive regions to a limited extent (Supplementary Figure 2D) and dramatically stained the area typically occupied by the Purkinje dendritic trees (Westenbroek et al., 1995). The antibody generated repeatable but faint bands in the high-molecular weight region corresponding to CaVs (Li et al., 2004) in Western blots of chick ghost lysate (Supplementary Figure 2A). One of these bands well-above 250 kDa is consistent with CaV2.2 while a second band located just below 250 kDa may correspond to chick CaV2.1 which typically runs at a lower molecular weight (Li et al., 2004). Western blots of Ab571 immunoprecipitated ghost lysate probed with C2Nt identified a strong band above 250 kDa, presumably CaV2.2 (data not shown).

### Compartment Analysis of Nanogold Clusters with the NmidC2 and C2Nt Antibodies

**FIGURE 7:**
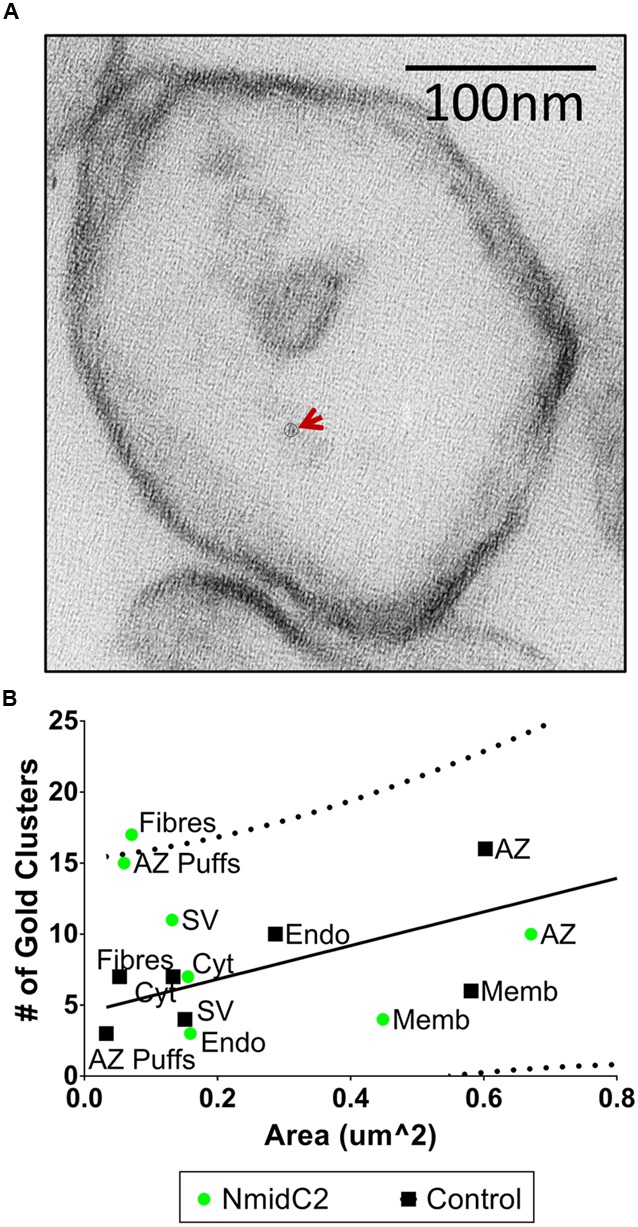
**Localization of the mid region of the channel C-terminal using the NmidC2 antibody. (A)** Synaptosome ghost immunogold labeled with NmidC2 with a nanogold cluster indicated by circle and arrow. **(B)** Compartment analysis of NmidC2, as in **Figures 4A,B** (*n* = 39, IgG control; *n* = 38, NmidC2 ghosts in 2 paired experiments).

**FIGURE 8:**
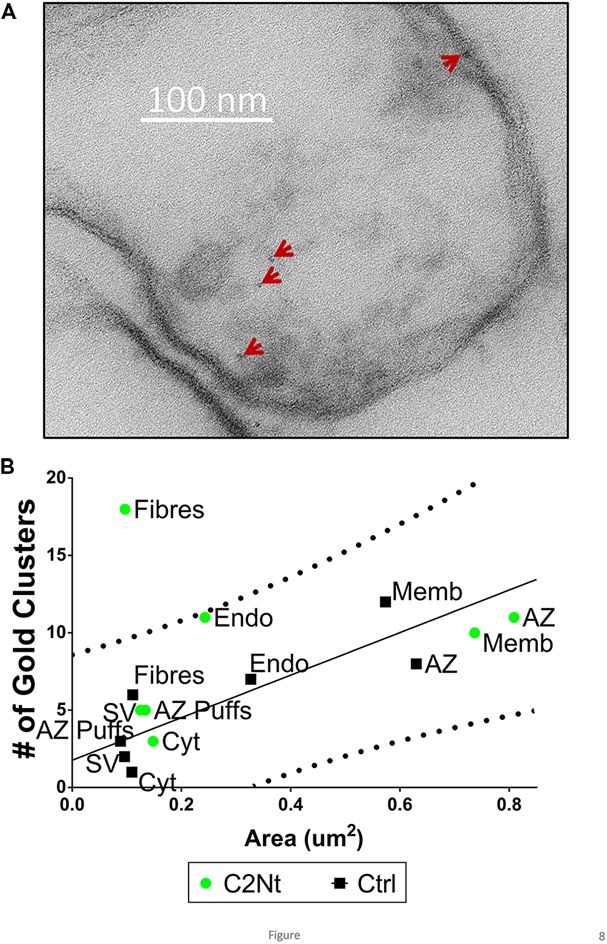
**Localization of the proximal region of the channel C-terminal using the C2Nt antibody. (A)** Synaptosome ghost immunogold labeled with C2Nt with nanogold clusters indicated by the arrows. **(B)** Compartment analysis of C2Nt, as in **Figures 4A,B** (*n* = 38, IgG control; *n* = 41, C2Nt ghosts in two paired experiments).

Synaptosome ghosts were labeled through cryoloading with affinity-purified NmidC2 (**Figure 7A**) or C2Nt (**Figure 8A**), as described above. With NmidC2, compartment analysis gold particle frequency was almost significant for AZ puffs, the electron dense ‘clumps’ on the AZ surface membrane (Phillips et al., 2001), but was significantly increased for AZ filaments (**Figure 7B**). All the other regions failed to reach significance.

Similar to the NmidC2 data and in contrast to the L45 data, C2Nt compartment analysis exhibited elevated nanogold clusters on AZ filaments but all other regions were within the control range (**Figure 8B**).

### Comparing Nanogold Cluster Location on Individual Tethered SVs with NmidC2 or C2Nt Antibodies to the C-Terminal Tip, L45 Antibody

**FIGURE 9:**
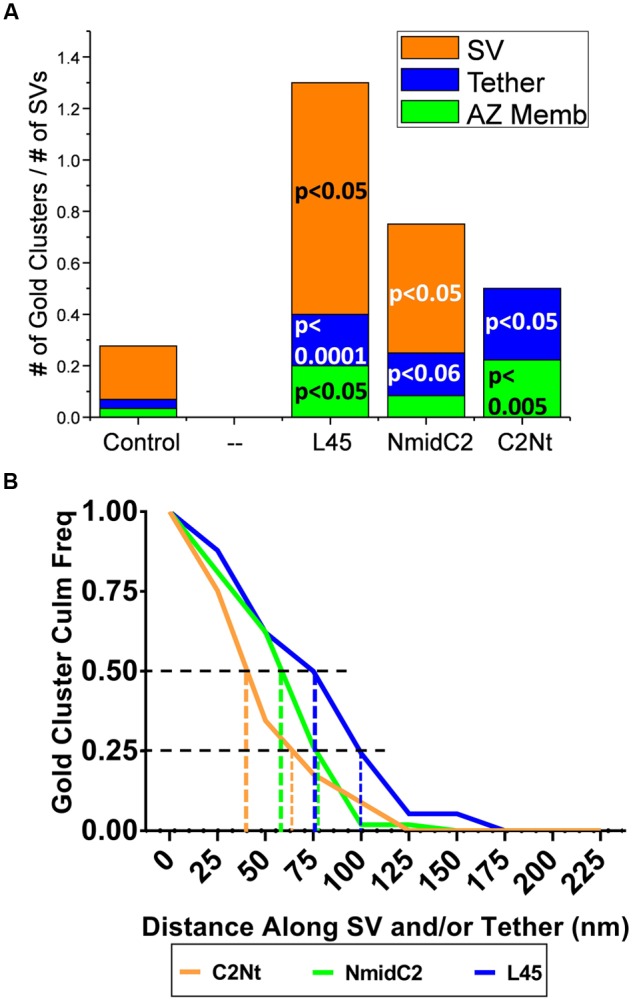
**Comparison of the distribution of nanogold clusters in individual tethered SVs for ghosts labeled with L45, NmidC2 and C2Nt. (A)** Individual tethered SVs from images of ghosts labeled with control (pooled for all three C-terminal immunogold labeling experiments), L45, NmidC2, or C2Nt antibodies were analyzed as in **Figures 5A,B**, dividing each tethered SV into AZ-membrane, fibrous tether and SV regions. (*n* = 55, 18, 12, and 10 for control, C2Nt, NmidC2 and L45, respectively). Test antibody data were analyzed against the pooled controls using the binomial tests with the *p* values shown on the plot. **(B)** Inverse cumulative frequency histogram of distances of gold clusters as traced along individual tethered SVs in ghosts immunogold labeled with C2Nt, NmidC2 or L45 antibodies. Data was compensated for non-specific labeling as measured with respective controls and normalized to the number of ghosts analyzed (*n* = 40, 38, and 12 for C2Nt, NmidC2, and L45, respectively). Vertical dashed and dotted lines denote the 50 and 75% nanogold cluster frequency distance, respectively, for each antibody condition.

If the CaV2.2 C-terminal tail extends from the AZ membrane to SVs in the interior of presynaptic terminals, then there should be distinct labeling patterns for the L45, NmidC2, and C2Nt antibodies along the channel C-terminal. As for the individual tethered SV analysis for L45 above, we counted the number of gold particles clusters that were associated with the SV, the tether, or the AZ membrane, attributing clusters within 10 nm of either the SV or the AZ membrane to those structures with the remainder counted as ‘on the tether.’ The gold particle cluster counts were normalized to the total number of individual tethered SVs imaged for each antibody type. With C2Nt, clusters were localized to the AZ membrane (4 clusters/18 tethered SVs) or to the tether itself (5 clusters/18 tethered SVs) but not to the SV. However, NmidC2 was similar to L45 with the majority of gold clusters associated with SVs (6 clusters/12 tethered SVs) and less with either tethers (2 clusters/12 tethered SVs) or the AZ membrane (1 cluster/12 tethered SVs) (**Figure 9A**).

When we measured the distances of gold particle clusters along tethers to the surface membrane (as we did above when comparing Ab571 to L45 labeling), we found that distribution of gold clusters for each of the C2Nt, NmidC2, and L45 antibodies (**Figure 9B**) relatively corresponded to the respective regions that the antibodies were targeted against (**Figure 1**). Half of the gold clusters from C2Nt-labeled tethered-SVs were within 45 nm from the surface membrane, while half of those from NmidC2- or L45-labeled tethered-SVs were within 60 and 80 nm away from the surface membrane, respectively. 75% of the gold clusters for C2Nt-, NmidC2-, or L45-labeled tethered SVs were within 65, 80, and 100 nm of the surface membrane, respectively.

### Antigenic Peptide-Blocked NmidC2 Antibody Eliminates Nanogold Labeling of Tethered SVs

As an additional control for specificity, we repeated the labeling procedures comparing immunogold labeling with NmidC2 sham-blocked with peptides corresponding to the antigenic region of L45 and the same antibody pre-blocked with the NmidC2 antigenic peptide. To focus on long tethers, we quantified nanogold clusters solely on SV tethers that were longer than 80 nm. As before, we counted gold clusters for <10 nm from the vesicle, <10 nm from the AZ membrane, and associated with the intervening tether. The number of gold particles was significantly higher in all three locations for the sham- than the pre-blocked antibody as tested by binomial (*p*_b_) analysis (AZ Membrane: *p*_b_ < 0.005; Tether: *p*_b_ < 0.005; SVs: *p*_b_ < 0.01).

## Discussion

The main findings of this study were that immunogold tagging of CaV C-terminals with three different antibodies resulted in nanogold clusters that are located not only on the AZ region of the surface membrane as expected, but also within the presynaptic cytoplasm associated with fibrous vesicular tethers. The key immunogold result was obtained with an antibody against the tip of the C-terminal, L45, which resulted in nanogold clusters on tethered SVs located within the presynaptic lumen at least as far as 100 nm from the AZ region of the surface membrane. We interpret these results to suggest that the channel C-terminal tail can bridge the gap between the AZ CaV and the SV.

Localization of the nanogold particles required mathematical analysis due to variability. This variability was attributed to the lengths of the primary antibody and Fab fragments, up to ∼15 nm from the primary antibody epitope. Further, our measurements do not take into consideration the z-axis displacement of structures within the 100 nm thick slices. While the antibody length error should be compensated by averaging, measurement errors in the *z*-axis might be predicted to always underestimate the actual distance. We did not attempt to correct for the latter.

This study follows from our earlier demonstration that SVs with short and long tethers can be revealed in electron micrographs after synaptosomes were ruptured by osmotic shock (Wong et al., 2014), a standard fractionation step used to isolate both SVs and synaptosome ghosts (Whittaker et al., 1964). In our preliminary studies, we used standard colloidal gold tagged secondary antibodies to localize our primary antibody, relying on diffusion and compromised plasma membranes for the antibodies to gain access to internal binding epitopes. While some gold labeling was observed, this was too infrequent to make definitive conclusions. To improve our signal to noise ratio, we made two key improvements to the gold labeling method. First, we used cryoloading (Nath et al., 2014) to freeze-load the synaptosome ghosts with the antibodies. Second, we replaced the conventional colloidal gold secondary antibody with Fab fragments that are covalently tagged with nanogold particles. With these improvements, the gold labeling was sufficiently abundant to carry out quantitative analysis (**Figure 2**). The only synaptosomes rejected for quantitative analysis were those that had resisted osmotic rupture during the wash steps and had passively trapped numerous nanogold Fab fragments.

The key finding of this study was that labeling with an antibody directed against the tip of the CaV2.2 C-terminal (L45) resulted in nanogold clusters associated with the residual, tethered SVs. This conclusion was supported by two different analysis methods: ‘compartment analysis,’ where we consider the distribution of gold clusters on all structures in the AZ region of the ghost, and direct examination of individual AZ-tethered SVs. This data argues that the CaV2.2 C-terminal tip localizes to the surface of SVs that are tethered to, but not necessarily abutting the AZ membrane (**Figures 4**, **5**). The simplest interpretation of these findings is that the channel C-terminal spans the gap between the AZ and the SV and is responsible for, or contributes to, SV tethering.

We also considered an alternative hypothesis to the C-terminal spanning the AZ to SV gap: that the entire CaV2.2 channel, that is the C-terminal as well the transmembrane regions, are all located on the SV. While proposed in one study (Zhang et al., 2000), this possibility seems unlikely as CaVs have not been reported on SVs using biochemical (Wong et al., 2013) or mass spectrographic analysis (Takamori et al., 2006). Nonetheless, we used two experimental strategies to test this idea. First, we used Ab571, an anti-CaV2.2 antibody that is directed against a peptide sequence in the II–III loop, to localize the channel using the same nanogold labeling approach. As expected, nanogold clusters were detected in the AZ membrane region. Surprisingly, we also detected Ab571 labeling of SVs (**Figure 6A**). Further analysis demonstrated that the nanogold clusters on the SVs were much closer to the AZ surface membrane with Ab571 (50% within 45 nm) than those observed with the C-terminal tip antibody, L45 (50% within 80 nm) (**Figure 6B**). Thus, this data does not support the idea that the channel pore is on the surface membrane but it does raise the intriguing possibility that the II–III loop (to which Ab571 binds) may also participate in SV tethering (see also below).

The second approach to testing if the channel C-terminal spans the gap between the surface membrane and the SV was to repeat the nanogold labeling using two new antibodies, NmidC2 and C2Nt, raised against peptides from central regions of the C-terminal. Note, however, that while L45 and NmidC2 are likely to be CaV2.2 specific, at least based on the antigenic AA sequence, C2Nt likely identifies both CaV2.1 and CaV2.2. Since these channels exhibit considerable homology and can substitute for transmitter release gating, we assume that their C-terminals share common functional domains. We predicted that if the channel pore is located on the SV itself with the entire C-terminal, the NmidC2- and C2Nt-associated nanogold clusters should have the same distribution as observed with L45. However, if the C-terminal spans the gap from the surface membrane to the SV, these clusters should be observed in the intervening cytoplasm in association with the fibrous tether. Overall, our observations favored the second conclusion.

The NmidC2 antibody was directed against an antigen approximately half way along the C-terminal. Using compartment analysis, NmidC2 significantly labeled filaments projecting from the AZ. While the frequency of nanogold clusters on SVs was higher than with the control pre-immune antibody, this did not reach significance according to compartment analysis (**Figure 7B**). However, with the more detailed individual tethered SVs analysis, the nanogold clusters were not only associated with the tether itself (*p* < 0.06), as expected, but there was also significant labeling of the tethered SVs (*p* < 0.05; **Figure 9A**). These results were replicated in a separate series of experiments comparing nanogold labeling using sham-blocked or antigenic peptide-blocked NmidC2 antibodies (See Results). However, a cumulative histogram analysis, comparing NmidC2 to L45, indicated that the nanogold clusters detected with the former were generally closer to the surface membrane (**Figure 9B**). Thus, the NmidC2 antigenic site is closer to the surface membrane than that for L45, consistent with the molecular structure of the channel C-terminal tail (**Figure 1**). One simple explanation for the association of the NmidC2 with SVs is that the C-terminal exhibits a second SV binding site in its mid-region in the vicinity of the NmidC2 binding site (see below).

We carried out a similar analysis using the C2Nt antibody, which was raised against a C-terminal peptide located approximately half way between the last transmembrane coil of CaV2.1 or CaV2.2 and the NmidC2 antigenic site (**Figure 1**). The findings with this antibody were unambiguous. Using the compartment analysis method, we only detected elevated nanogold clusters on the cytoplasmic fibers that project from the AZ (**Figure 8B**), which we assume include un-occupied SV tethers. Further, analysis of individual tethered SVs exhibited a highly significant elevated nanogold cluster frequency on the tethers and near the AZ membrane, while no clusters were associated with the tethered SVs themselves (**Figure 9A**). Thus, the C2Nt result provides additional evidence against the idea that the channel pore is located on the SV and support the hypothesis that the C-terminal spans the AZ-SV cytoplasmic space.

Overall, our results are consistent with the hypothesis that the AZ CaV2 channel can extend its C-terminal into the nearby cytoplasm and that its tip, and likely at least one other point along its length, can attach to SVs up to ∼180 nm away. Fibrous linkers that extend from the AZ region of the surface membrane and contact SVs have been reported in a number of previous studies (Landis et al., 1988; Hirokawa et al., 1989; Siksou et al., 2007; Fernández-Busnadiego et al., 2013; Wong et al., 2014; Cole et al., 2016) but little is known about their molecular composition. Using a cell-free system, we recently reported that SVs can be captured by the intact CaV2.2 channel and can bind to a fusion protein comprising the distal half to a third of its long C-terminal (Wong et al., 2013). Further analysis restricted the binding site to a 51 aa sequence just proximal to the C-terminal tip (Wong et al., 2014) and we recently identified a 5 AA putative SV-binding motif (Gardezi et al., 2016). We speculated that if, as predicted by sequence software, the long C-terminal is mostly unstructured, its backbone could extend as far as 200 nm from the surface membrane. Fibrous linkers between tethered SVs and the surface membrane that extended up to this limit were observed in synaptosome ghosts (Wong et al., 2014). The current findings add support to the idea that these linkers can comprise, or at least contain, the channel C-terminal and stimulate some provocative models with respect to SV capture from the cytoplasm and the subsequent docking steps.

The statistical significance of our nanogold localization analysis argues that this putative mechanism of C-terminal tethering is fairly common but further analysis will be necessary to determine if such a link plays a role in SV recycling. Our current working model hypothesizes two types of channel-SV links that we termed G- (Grab-) and L- (Lock-) tethers (Wong et al., 2014). We speculate that at least for the CaV2.2 channel, a single long G-tether serves to capture an SV from the cytoplasmic pool and serves as a guide to bring the SV toward its docking site. Once close to the CaV (< ∼45 nm), additional tethering occurs, as supported by the visualization of multiple short tethers (Fernández-Busnadiego et al., 2013; Wong et al., 2014; Cole et al., 2016). A requirement for a second, L-tethering mechanism was dictated by the need to bring the docked SV within range of the single channel Ca^2+^ domain (Fogelson and Zucker, 1985; Simon and Llinás, 1985; Weber et al., 2010), estimated from functional and structural studies to be ∼25 nm (Stanley, 1993, 1997, 2016; Weber et al., 2010; Dittrich et al., 2013). Based on earlier work that reported that the exocytosis-related protein Syntaxin 1A can bind to a characterized ‘synprint’ sequence on the channel II–III loop (Sheng et al., 1998; Catterall, 1999; Atlas, 2001) and the effect of block of this interaction (Mochida et al., 1996), we speculated that this link might account for L-tethering. Our findings support this hypothesis in that nanogold clusters localized by Ab571, which binds to the synprint sequence, also associated with short-tethered SVs. NmidC2 nanogold clusters were closer to the surface membrane than those labeled by the C-terminal tip antibody L45 (**Figure 9B**), supporting our hypothesis that the C-terminal spans the surface-membrane-to-SV cytoplasm. Interestingly, these clusters were also associated with SVs (**Figure 9A**). This was unlikely to reflect a curious artifact since nanogold clusters observed with C2Nt, which binds to the C-terminal closer to the surface membrane (**Figures 1**, **9B**), were not associated with SVs (**Figures 8B**, **9A**). Thus, the observations with NmidC2 argue for a second SV binding site in the mid-region of the C-terminal mid-region and up to 100 nm from the surface membrane (**Figure 1**). Such a site could also contribute to SV alignment to the CaV pore during docking in preparation for single domain gating.

## Ethics Statement

Only pre-viable chick embryos were used in this study which are not subject to ethics approval.

## Author Contributions

Conceived project: ES and RC. Carried out electron microscopy preparation and imaging, immunogold staining, dot blots; data analysis: RC. Conceived gold particle localization statistical tests: ES. Created fusion protein constructs for dot-blots: QL. Carried out biochemical analyses including Western blots and IP: RC, SG, and CS. Carried out immunostaining: RC and QL. Wrote MA: RC and ES. Funded project and project responsibility: ES.

## Conflict of Interest Statement

The authors declare that the research was conducted in the absence of any commercial or financial relationships that could be construed as a potential conflict of interest.
