# Supplementary material for: The Calcium Channel C-Terminal and Synaptic Vesicle Tethering: Analysis by Immuno-Nanogold Localization
Source: Front Cell Neurosci. 2017 Mar 30;11:85. doi: 10.3389/fncel.2017.00085 (PMC5371611; doi:10.3389/fncel.2017.00085)

***Supplemental Figure 1.*** **Characterization of NmidC2 Antibody. (A)** ***Western blot characterization of NmidC2.*** *Left panel*: Western blots of ghost lysate probed with pre-immune serum (IgG), NmidC2 antibody, or Ab571 antibody. Arrow denotes the band corresponding to CaV2.2 as identified by the previously characterized Ab571 antibody. *Right panel:* Western blot of fusion proteins probed with NmidC2 antibody. The C1-2 and CmidA fusion proteins span the mid-region and contain the NmidC2 antigenic site whereas the CproxA fusion protein terminates before the antigenic site. Arrows denote the band location of each fusion protein, based on molecular weight and parallel GST blots (all fusion proteins are GST tagged; data *not shown*). **(B)** ***Dot blot characterization of NmidC2.*** The panel shows a single transfer sheet with 10 trials (rectangular boxes) each with four immobilized fusion proteins (coloured circles). In each box the upper fusion protein pair is C3, which contains the L45 binding site but lacks that for NmidC2, while the lower pair is C1-2 fusion protein that contains the NmidC2 binding site but lacks that for L45. As indicated, for each pair the right fusion protein dot contains ten times the amount of fusion protein as its partner on the left. The immobilized fusion proteins were probed with either L45 (*top row*) or NmidC2 (*bottom row*) antibodies that were pre-treated with peptides as indicated. The peptide used to raise the antibody was used to block L45 or NmidC2 and the reciprocal peptides were used for sham-blocks. **(C)** ***Immunocytochemistry characterization of NmidC2.*** Dissociated chick ciliary ganglion calyx-synapse neurons were immunostained with SV2 (magenta), an SV marker, to identify the calyx presynaptic terminals, and NmidC2 (green). Note the punctate transmitter release face staining with the latter.

***Supplemental Figure 2.*** **Characterization of C2Nt Antibody. (A)** ***Western blot of ghost lysate probed with pre-immune serum (IgG) or C2Nt***. Arrows identify high-molecular weight bands that are in the right region for CaVs. **(B)** ***Fusion protein characterization of C2Nt.*** C1-2 and CproxA fusion proteins (see ***Supplemental Fig. 1A***) contain the C2Nt antigenic site. CmidB fusion protein replicates a middle region of the CaV2.2 C-terminal that is distal to the C2Nt antigenic site. C5 is a fusion protein replicating the CaV2.1 C-terminal and contains the C2Nt antigenic site. Arrows denote the location of each fusion protein as predicted by molecular weight and identified by anti-GST (*all fusion proteins are GST tagged; data not shown*). **(C)** ***C2Nt immunostain of the chick ciliary ganglion calyx synapse.*** Preparation as in ***Supplemental Fig. 1C.*** SV2 staining (magenta) identifies SV clusters within the calyx presynaptic terminal. C2Nt immunostain (green) resulted in punctate staining of the calyx transmitter release face, consistent with CaV clusters. **(D)** ***Chick cerebellar slice immunostained with Calbindin and C2Nt.*** Calbindin staining (magenta) was used to identify Purkinje cell bodies but in E15 chick the staining did not extend into the dendritic tree. C2Nt resulted in moderate staining of the calbindin-positive neuron somatic membrane. More extensive staining was observed on long structures within the region that contains Purkinje dendritic ‘trees’ (region above the Purkinje soma layer), although we do not know what these structures are, and likewise within the deeper granular layer (region below the Purkinje soma layer).

**Supplemental Figure 1.**

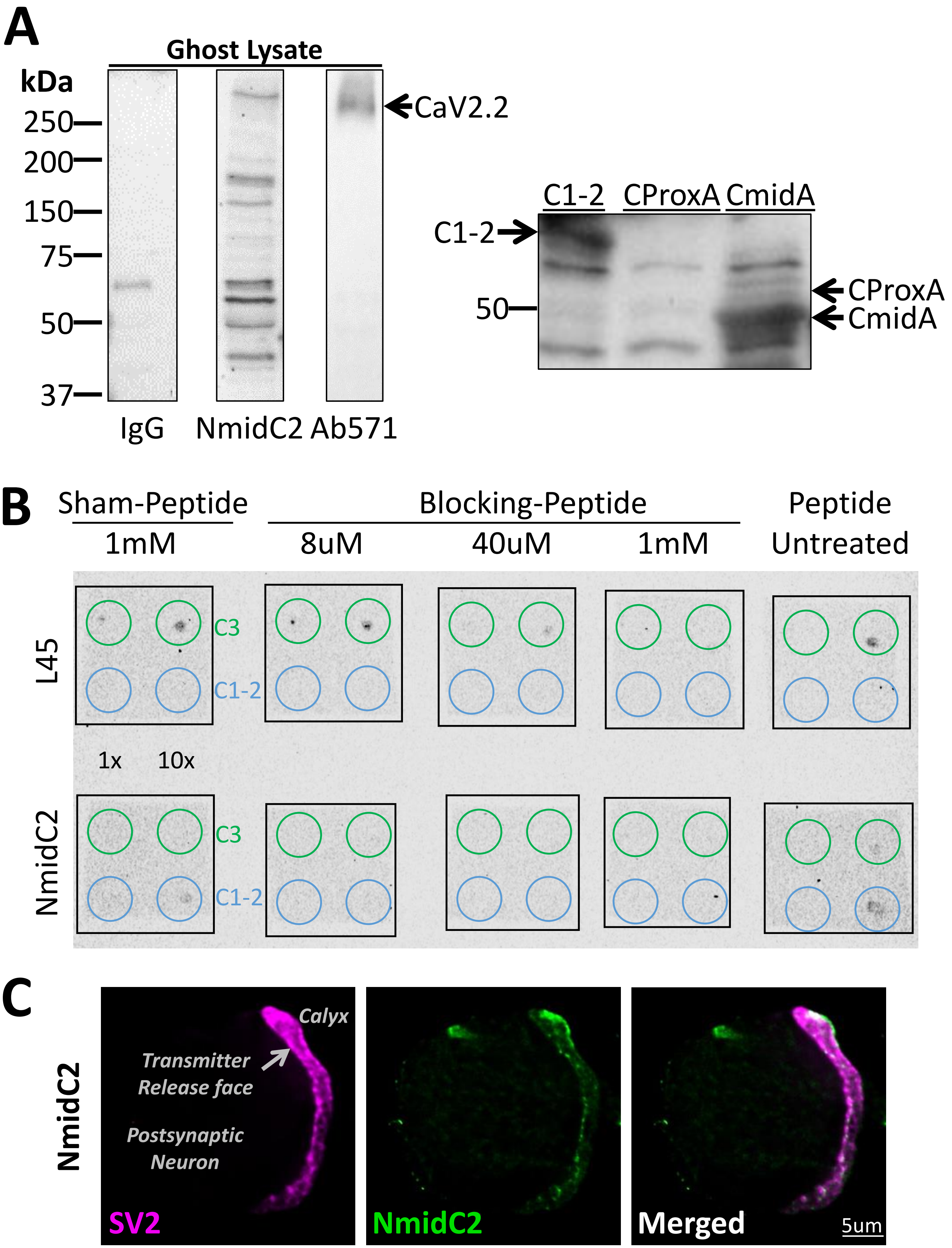

**Supplemental Figure 2.**

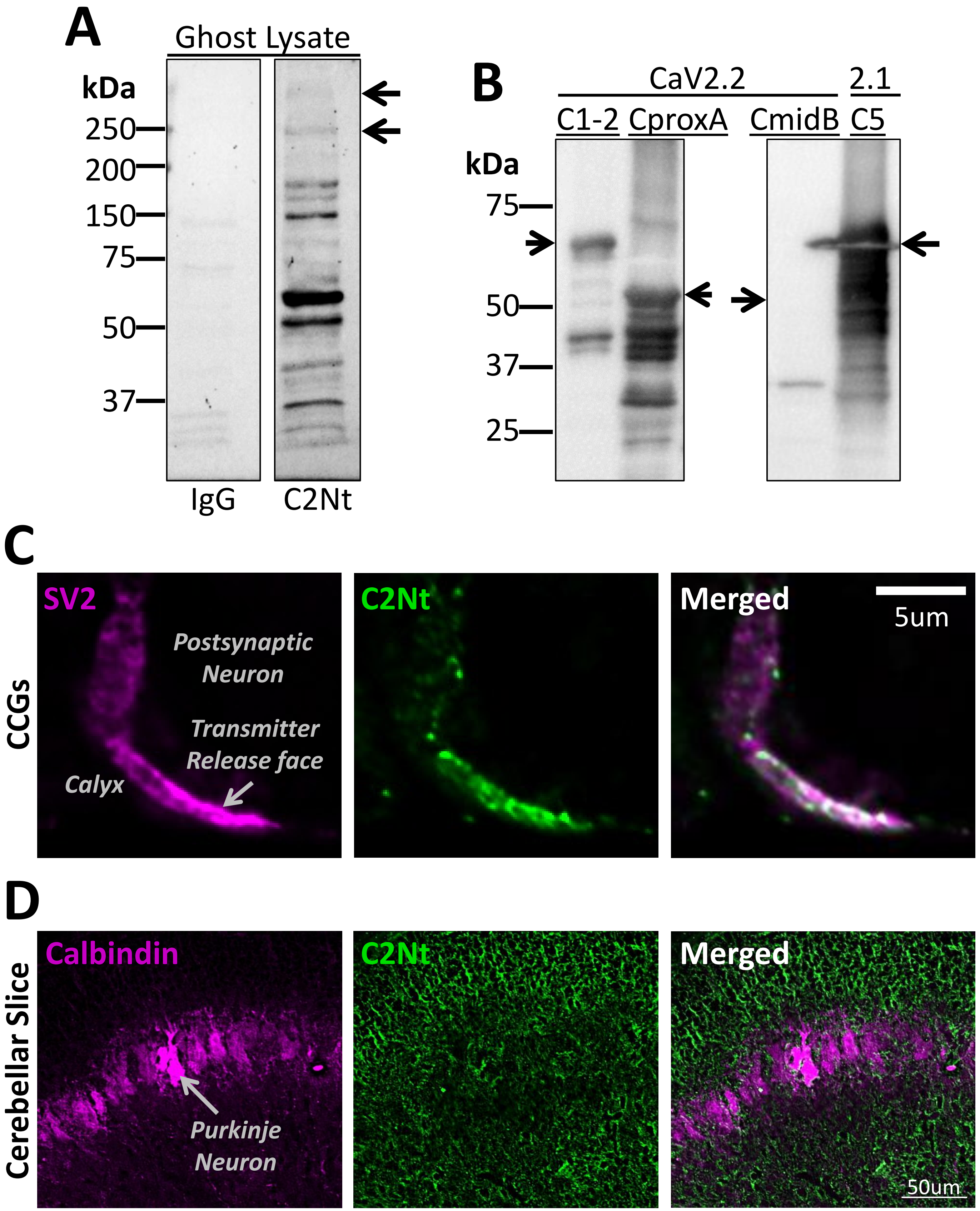

Supplement: Supplementary file 1 [file Image_1.PDF]
